# *In-Situ* Electron Channeling Contrast Imaging under Tensile Loading: Residual Stress, Dislocation Motion, and Slip Line Formation

**DOI:** 10.1038/s41598-020-59429-x

**Published:** 2020-02-14

**Authors:** Keiichiro Nakafuji, Motomichi Koyama, Kaneaki Tsuzaki

**Affiliations:** 1 Department of Mechanical Engineering, Kyushu University, Motooka 744, Nishi-ku, Fukuoka 819-0395 Japan; 2 Institute for Materials Research, Tohoku University, 2-1-1 Katahira, Aoba-ku, Sendai, Miyagi 980-8577 Japan; 3 Elements Strategy Initiative for Structural Materials (ESISM), Kyoto University, Yoshida-honmachi, Sakyo-ku, Kyoto 606-8501 Japan

**Keywords:** Mechanical properties, Metals and alloys

## Abstract

Elastoplastic phenomena, such as plastic deformation and failure, are multi-scale, deformation-path-dependent, and mechanical-field-sensitive problems associated with metals. Accordingly, visualization of the microstructural deformation path under a specific mechanical field is challenging for the elucidation of elastoplastic phenomena mechanisms. To overcome this problem, a dislocation-resolved *in-situ* technique for deformation under mechanically controllable conditions is required. Thus, we attempted to apply electron channeling contrast imaging (ECCI) under tensile loading, which enabled the detection of lattice defect motions and the evolution of elastic strain fields in bulk specimens. Here, we presented the suitability of ECCI as an *in-situ* technique with dislocation-detectable spatial resolution. In particular, the following ECCI-visualized plasticity-related phenomena were observed: (1) pre-deformation-induced residual stress and its disappearance via subsequent reloading, (2) heterogeneous dislocation motion during plastic relaxation, and (3) planar surface relief formation via loading to a higher stress.

## Introduction

Metals have been extensively used in structural applications due to their superior ductility and toughness. These characteristics are attributed to plasticity-driven strain evolution and associated stress re-distribution. The plasticity is originated from dislocation glides, which have a variable behavior sensitive to local shear stress, depending on external load and stress concentration. Therefore, in order to understand ductility and toughness, the plasticity mechanisms under certain mechanical conditions need to be shown. In this context, observations using a dislocation-resolved *in-situ* technique and done under mechanically controllable conditions are the most suitable approach.

From a spatial resolution perspective, transmission electron microscopy (TEM) is an effective technique for resolving dislocations^1–4^. However, regarding *in-situ* deformation experiments, the special boundary conditions in a thin foil-type specimen may be problematic, giving unwanted signals such as effects of image force^5–9^. Consequently, in addition to the TEM approach, there is a high demand for a dislocation-resolved *in-situ* technique suitable for bulk specimens.

Electron channeling contrast imaging (ECCI) enables visualization of dislocations^10–13^, stacking faults^6,11,12,14^, twins^11,12,15,16^, and elastic strain fields using a field-emission scanning electron microscope. Thus, ECCI can be used to characterize bulk specimens, allowing for the use of the conventional geometry of the mechanical test specimens^13,17–19^. Therefore, dislocation-resolved ECCI was chosen as a mechanically specified *in-situ* technique for the analysis of deformation. Moreover, since lattice defects can be detected under optimal specimen surface orientation, nanometer-scale deformation heterogeneity can be visualized in an observational area greater than a 10 µm square.

Therefore, in this study, we investigated the suitability of ECCI as an *in-situ* characterization technique, while presenting its applicability for *in-situ* observation of a bulk metallic specimen under tensile loading.

## Results and Discussion

### Visualization of residual stress variation

**Figure 1:**
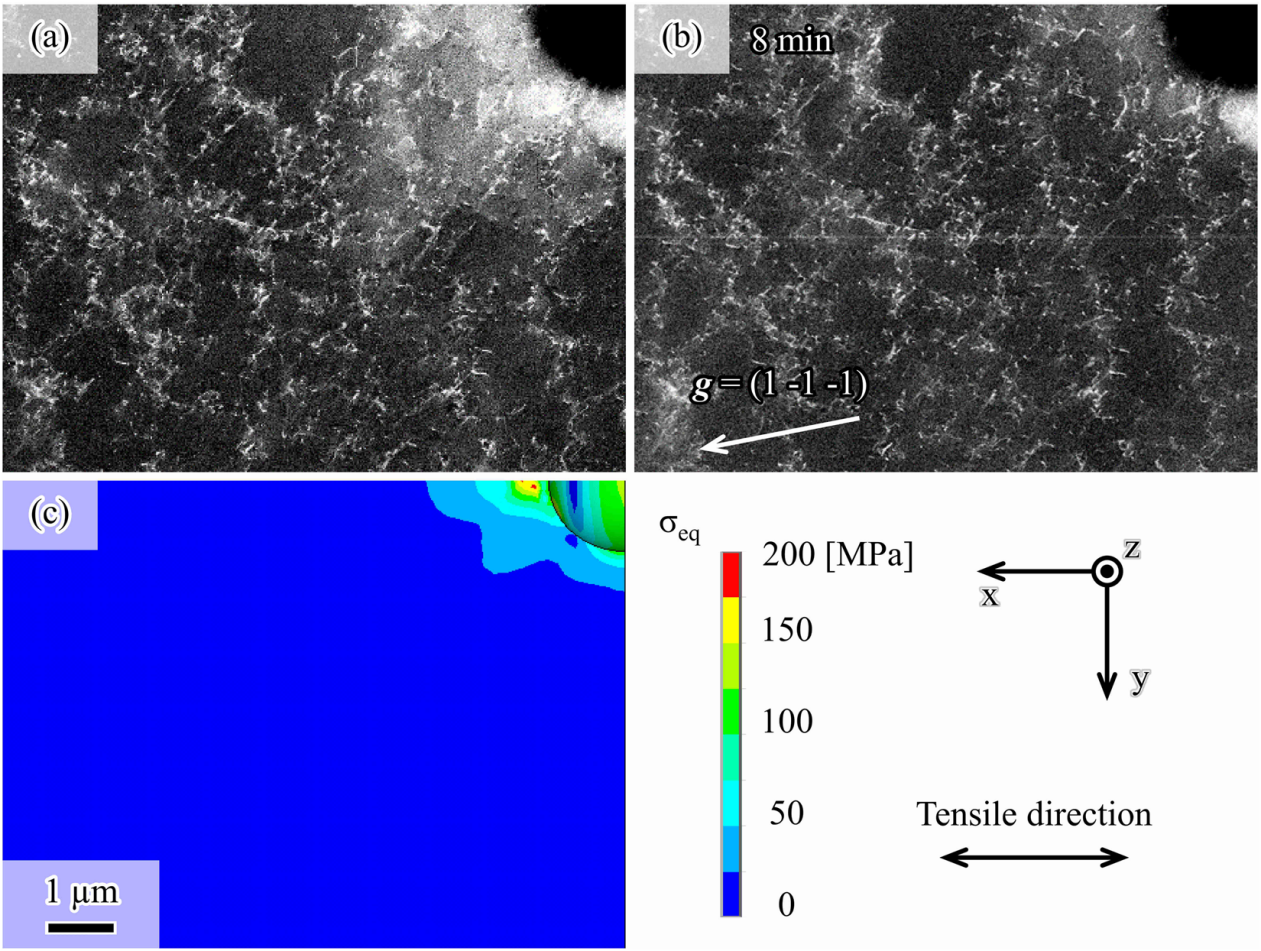
Change in electron channeling contrast associated with relaxation of residual compressive stress. Electron channeling contrast images of the 2% pre-deformed and mechanically polished specimen (**a**) under an unloading condition and (**b**) after reloading to 199 MPa and displacement holding for 8 min. The incident beam direction was [0.51 –0.35 0.79] near the [1 –1 2] direction. The initial 1-μm-radius circular hole corresponds to the black areas in the upper right of the images. (**c**) von Mises equivalent stress (σ_eq_) map of the pre-deformed specimen without external stress. The general-purpose finite element program ANSYS 17.0 (http://www.ansys.com/) was used to depict (**c**).

Figure 1a shows an electron channeling contrast (ECC) image without external stress. This image was taken after the sample was submitted to pre-deformation of 2% tensile strain (corresponding to 246 MPa of external stress), followed by mechanical polishing. The alloy used in this experiment was an fcc ferrous alloy with the chemical composition of Fe15Mn10Cr8Ni in mass%. The loading and reloading process is shown in Fig. S1. As shown in Fig. 1a, a wide white area appears around the circular hole, corresponding to the black area in the upper right side. Additionally, Fig. 1b shows the microstructure with external stress after reloading to 199 MPa (elastic regime) and subsequent displacement holding for 8 min. As seen, the white area disappears after the reloading in the elastic regime. Note that significant dislocation movement was not observed during the reloading, as recognized by comparing Fig. 1a,b. Thus, the change in the electron channeling contrast from Fig. 1a,b indicates that the local contrast difference around the circular hole in Fig. 1a arises from the presence of residual stress.

To clarify the origin of the white region in Fig. 1a, we utilized a three-dimensional finite element method and found that residual stress exists around the circular hole after 2% pre-straining and unloading (Fig. 1c). The 2% pre-straining causes significant plastic strain and higher strain near the circular hole due to the stress concentration. Thus, the region surrounding the stress concentration source consequently compresses the region with high plastic deformation during the unloading process. Therefore, the elastic deformation attributed to the residual compressive stress produces elastic lattice strain and lattice rotation.

Furthermore, the electron channeling contrast has a strong dependence on the backscatter electron signal because of the electron channeling mechanism. This signal is originated from the angular difference of the incident beam, starting from a Bragg position, with respect to low-index lattice planes, which are nearly parallel to the beam. Hence, the lattice strain and lattice rotation change the angular difference, and consequently, the electron channeling contrast.

Therefore, the white region in Fig. 1a originated from the residual compressive stress. In turn, tensile reloading relaxes this residual compressive stress, resulting in the disappearance of the white region observed in Fig. 1b. To our knowledge, this is the first successful report of residual stress variation visualization using ECCI.

### *In-situ* observation of dislocation motion

**Figure 2:**
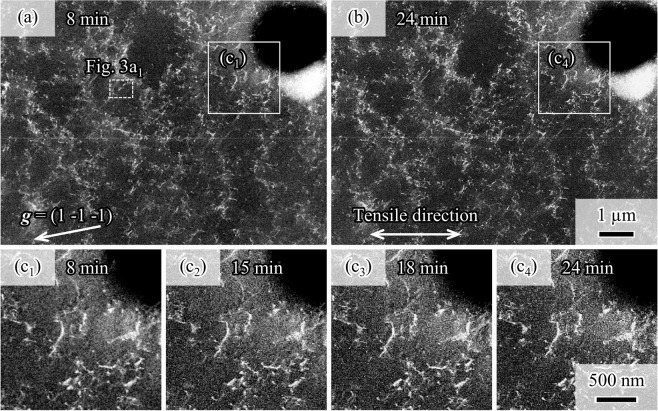
Dislocation distribution around the circular hole during the displacement holding test performed after reloading to 199 MPa (elastic regime). (**a**) Overview of the area observed using ECCI, after displacement holding for 8 min. (**b**) ECC image after displacement holding for 24 min. (c_1_)–(c_4_) Sequential ECC images of the area marked by white lines in (**a,b**) after displacement holding for (c_1_) 8, (c_2_) 15, (c_3_) 18, and (c_4_) 24 min. A movie of (**c**) is available in the supplemental materials.

Next, we determined the microstructure-dependent heterogeneous motion of dislocations. As previously discussed, the region near the hole was preferentially deformed due to stress concentration. However, dislocations around the hole were not observed during the displacement holding test conducted after reloading to 199 MPa (shown in the area marked by white lines in Fig. 2a,b). Moreover, Fig. 1 showed a compressive residual stress around the hole after the 2% straining and unloading. Therefore, we hypothesize that the compressive residual stress decreases the local resolved shear stress produced by the reloading, which, in turn, reduces the driving force for dislocation motion near the hole. This was supported by the lack of evidence of dislocation motion near the circular hole area (seen in Fig. 2c_1_–c_4_).

**Figure 3:**
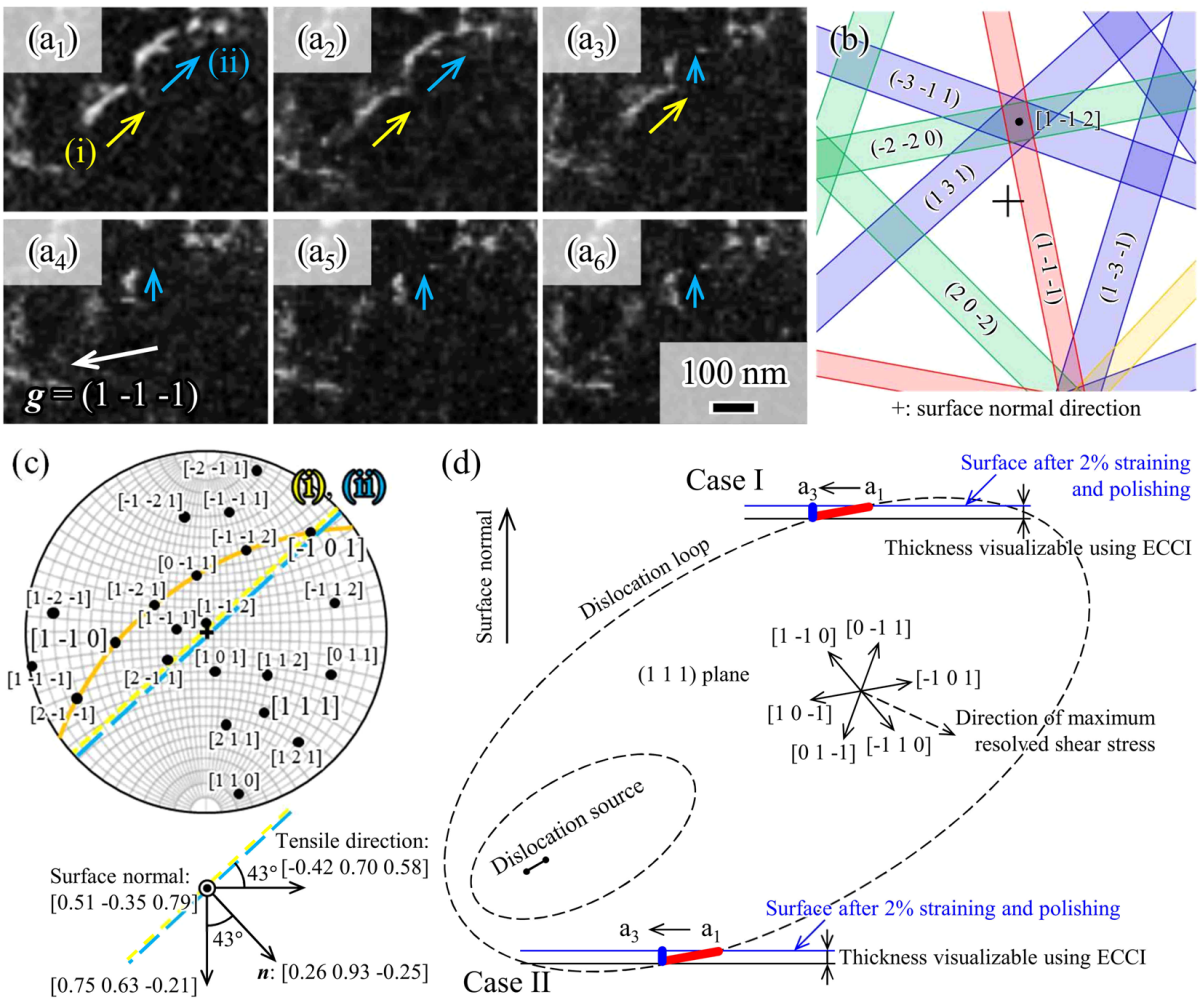
Dislocation motions in the area marked in Fig. 2a, after reloading to 199 MPa and displacement holding for (a_1_) 8, (a_2_) 11, (a_3_) 15, (a_4_) 18, (a_5_) 21, and (a_6_) 24 min. (**b**) Simulated electron channeling pattern with the incident beam direction (i.e., [0.51 –0.35 0.79]). (**c**) Stereographic projection representing <1 1 1>, <1 1 0>, and <1 1 2> directions, where the yellow and green dashed lines indicate the traces of dislocations (i) and (ii) in (a_1_). The orange curve indicates the (1 1 1) plane as a great circle. (**d**) Schematic dislocation loop indicating the possible two cases of dislocation segment (ii) in (a_1_). A movie of (**a**) is available in the supplemental materials.

Contrastingly, we found dislocation motions during the displacement holding in regions considerably away from the hole (shown as Figs 3a_1_ in 2a). Figure 3a_1_–a_6_ show sequential ECC images of the area marked by the white dashed lines in Fig. 2a after reloading to 199 MPa (elastic regime) and subsequent displacement holding for 8, 11, 15, 18, 21, and 24 min, respectively. These figures picture two different dislocations on the {1 1 1} planes of the fcc ferrous alloy. It is possible to see that these dislocations ((i), yellow arrows and (ii), blue arrows) moved during the holding. Notably, dislocation motion was not observed when the stress level was low as shown in Fig. 2c_1_–c_4_. That is, the electron beam during the imaging did not induce any dislocation motion under the present observational condition. In other words, the dislocation motion shown in this study was attributed to external stress effects. Also, since the displacement holding was conducted at an ambient temperature, we concluded that these movements were originated from a glide process and not from a climb process.

Thus, we tried to identify the activated slip system of the dislocations. Unfortunately, the resulting Burgers vectors could not be determined through g·b = 0 analysis, due to the conditions used for the incident beam direction - [0.51 –0.35 0.79] near the [1 –1 2] pole (Fig. 3b). With this beam direction, we could not obtain {1 1 1} or {0 0 2} diffraction vectors that had sufficiently strong backscattered electron intensity for ECCI except for the (1 –1 –1) vector. Therefore, we used the Schmid factors in order to estimate the slip system (summarized in Table 1). According to Schmid factor criteria, the slip plane where the dislocations glided was the (1 1 1) plane, with a slip direction of [−1 0 1] or [1 –1 0]. With this information, we then examined the dislocation characteristics based on the dislocation morphology. The dislocation line directions shown in Fig. 3a_1_ were estimated from both the slip plane and the dislocation line traces in the stereographic projection (shown in Fig. 3c). Any dislocation line trace was obtained through the projection trace of partial and perfect dislocations on the specimen surface. Note that the line traces of dislocations (Fig. 3a(i,ii)) are parallel, and that the angle between the line trace and the tensile direction [−0.42 0.70 0.58] is 43°. Thus, the dislocations are on the plane of which the normal direction is ***n***, as shown in the following formula:

1 $$n=[\,-0.42\,\,0.70\,\,0.58\,]\,\sin \,{43}^{\circ }+[\,0.75\,\,0.63\,\,-0.21\,]\,\cos \,{43}^{\circ }=[\,0.27\,\,0.94\,\,0.24\,]$$

**Table 1:** Schmid factors of the slip system for the observed grain. The incident beam direction was [0.51 –0.35 0.79] and the tensile direction was [−0.42 0.70 0.58].

Slip plane	Slip direction	Schmid factor
(1 1 1)	[0 1 –1]	0.04
	[−1 0 1]	0.35
	[1 –1 0]	0.39
(−1 1 1)	[0 –1 1]	0.08
	[−1 0 –1]	0.11
	[1 1 0]	0.19
(1 –1 1)	[0 1 1]	0.28
	[1 0 –1]	0.22
	[−1 –1 0]	0.06
(1 1 –1)	[0 –1 –1]	0.16
	[1 0 1]	0.02
	[−1 1 0]	0.14

([0 42 0 70 0 58]: right direction in Fig 3c, [0 75 0 63 0 21] : downward direction in Fig 3c).

The dislocations are also on the (1 1 1) plane from the Schmid factor discussion and hence the dislocation line vector ***t*** can be determined as follows:

2 $$t=[\,1\,\,1\,\,1\,]\times n=[\,-0.69\,\,0.02\,\,0.67\,]$$

When the dislocations have a ± a/2[−1 0 1] Burgers vector, the angle between the Burgers vector and the dislocation line vector ***t*** is 2° and they have a screw character. When the dislocations have a ± a/2[1 –1 0] Burgers vector, the angle between the Burgers vector and the dislocation line vector ***t*** is 122° and they have a mixed character.

Once the dislocation line vector was determined, the visible depth for ECCI could be evaluated. When the dislocation line vector ***t*** is given as [−0.69 0.02 0.67] in Eq. (2), the visible depth in the sample becomes a value between 20 and 30 nm using the line lengths of the projected dislocations (130 nm) and the inclination angles of the dislocation lines with respect to the surface (10°). The typical value of the maximum depth of visibility has been reported to vary between 50 and 100 nm for scanning electron microscope conditions^11^, which is greater than the range obtained in our study. However, the range difference could come from the sample used in the reported study containing sufficient chromium (10 mass%) to form a thin passive film of chromium oxide, leading to a maximum depth of visibility smaller than normally expected.

Next, we examined the validity of the dislocation motion observed during the displacement holding test performed after reloading to 199 MPa (elastic regime) (Fig. 3a). The dislocations in Fig. 3a_1_ are either segment (I) or (II) of the dislocation loops, schematically depicted by the red lines in Fig. 3d. Note that the specimen was pre-deformed to a 2% tensile strain and then mechanically polished with a layer of 30 µm in thickness. Hence, the dislocation source was considered to either be above or below the dislocation segment. Case I shows that the source still exists in the specimen (below the dislocation segment), while case II indicates that the source was in the polished region (above the segment). Fig. 3a_3_–a_6_ depict dislocation (ii) as a dot, indicating that the dislocation line changed to be approximately perpendicular to the specimen’s surface. With this position change, the dislocation loop shrinks in case II, whereas in case I the loop expands for the present dislocation motion. Note that the dislocation loops must expand for plastic relaxation during the displacement holding. Thus, it is provable that segment (ii) observed in Fig. 3a_1_ corresponds to case I in the schematic of Fig. 3d. It is also supported by the length of the dislocation line. When the dislocation line is approximately perpendicular to the surface, namely the line length of the projected dislocation is smallest on the (1 1 1) plane, the value of the inclination angle of the dislocation line with respect to the surface is 33° and the value of the line length of the projected dislocation is between 10 and 20 nm. This is reasonable for the expanding motion of the dislocation loop, with the dislocation source below the segment.

However, the image force from the specimen surface was not considered in the aforementioned discussion. To evaluate the image force in a simple manner, it was assumed that dislocation (ii) had a screw character with a [−1 0 1] Burgers vector and the distance between dislocation (ii) and the image dislocation was 40 nm, corresponding to twice the visible depth for ECCI (20–30 nm). With this assumption and a shear modulus of G = 78.9 GPa^20^, we obtained shear stress of 68 MPa in the [−1 0 1] direction on the (1 1 1) plane, which arises from the image dislocation. The magnitude was approximately the same as the resolved shear stress arising from the external load: 70 MPa (199 MPa × 0.35). Therefore, when considering the image force, case II in the schematic of Fig. 3d is also possible for the case of dislocation segment (ii) shown in Fig. 3a_1_.

### *In-situ* observation of slip line formation

**Figure 4:**
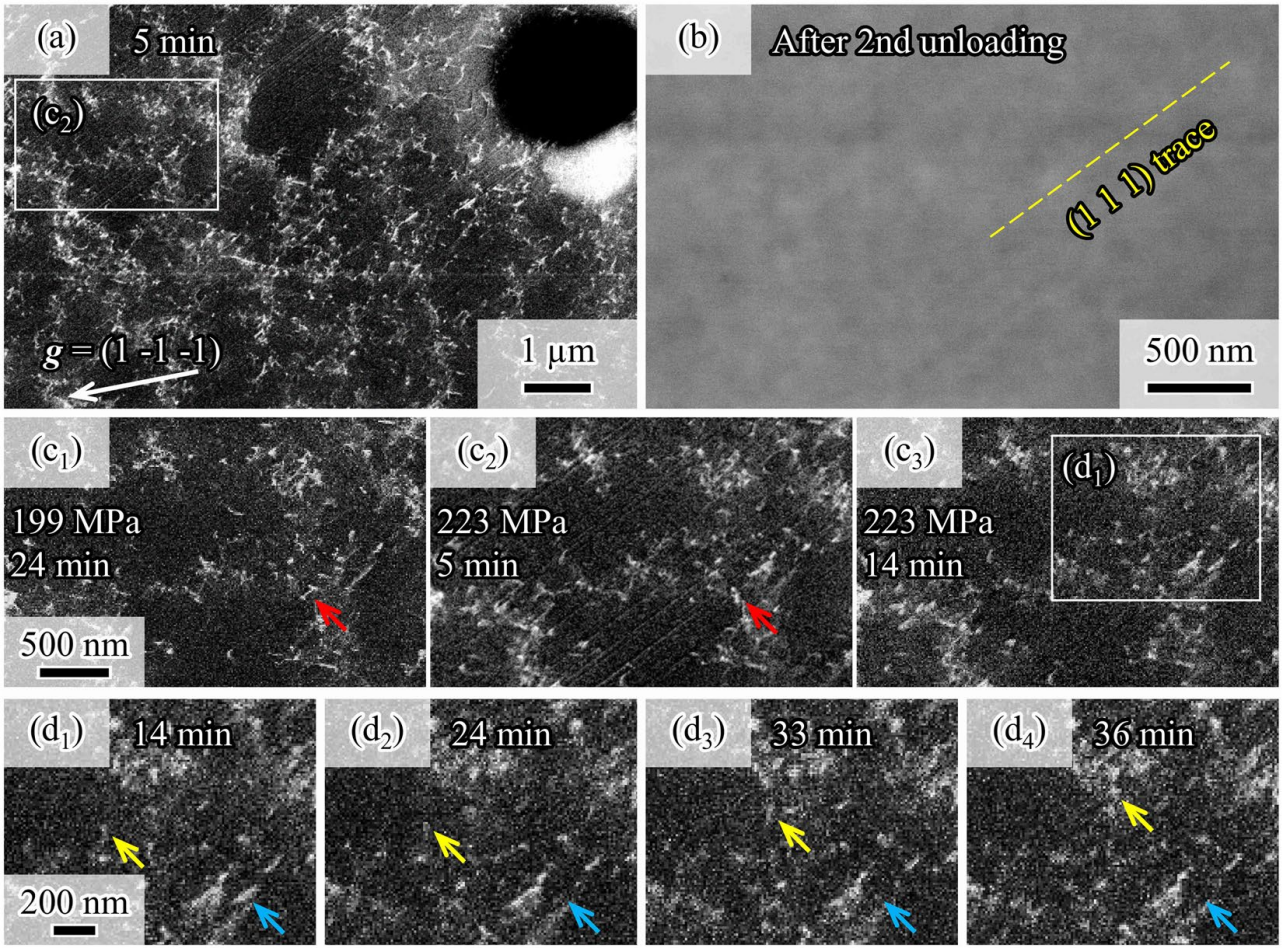
Formation of line patterns and dislocation motions during the displacement holding test performed after further loading to 223 MPa (elastic regime). (**a**) ECC image depicting nearly the same area shown in Fig. 3a. (**b**) Secondary electron image of the area marked in (**a**) taken after the second displacement holding and unloading. This image was obtained at an acceleration voltage of 10 kV, a probe current of 10 nA, and a working distance of 10.3 mm. (c_1_)–(c_3_) ECC images of the area in (**b**). (c_1_) was obtained after loading to 199 MPa and displacement holding for 24 min. (c_2_) and (c_3_) were obtained after further loading to 223 MPa and displacement holding for 5 and 14 min, respectively. The red arrows show the shape change of the dislocation line in a high dislocation density wall. (d_1_)–(d_4_) ECC images of the area marked in (c_3_) after loading to 233 MPa and displacement holding for (d_1_) 14, (d_2_) 24, (d_3_) 33, and (d_4_) 36 min. Movies of Fig. 4c,d are available in the supplemental materials (Figs. S5 and S6). Wider region figures of Fig. 4 are available in Fig. S7.

After displacement holding for 24 min (Fig. 3a_6_), the specimen was further loaded to 223 MPa, with the loading stage still macroscopically elastic, and held at constant displacement for 281 min (Fig. S1). After the second displacement holding and unloading, a new type of defect, line patterns, was determined using secondary electron imaging (shown in Fig. 4b, corresponding to the area marked by white lines in Fig. 4a). Secondary electron imaging is a surface-relief-sensitive technique. Hence, the observed line patterns were attributed to surface relief, (i.e. slip lines). The observed lines are along the (1 1 1) plane, which is the slip plane with the maximum Schmid factor (Table 1). Note that the angle between the specimen surface and the (1 1 1) plane is 57°. Similar line patterns were not observable using ECCI during the stage after reloading to 199 MPa and displacement holding for 24 min (Fig. 4c_1_). However, these patterns were observed after further loading to 223 MPa and displacement holding for 5 min (Fig. 4c_2_), indicating that these line patterns were induced by a stress increase.

Notably, Fig. 4 shows that both motion and morphology are different between the single dislocations and line patterns. For single dislocations, there are two motion patterns depending on the dislocation density. In the region of a high dislocation density wall, significant movement was hardly observed, but a shape change in the dislocation line was determined (red arrows in Fig. 4c_1_–c_2_). However, some single dislocations show significant movement in cell core regions with a low dislocation density (yellow and blue arrows in Fig. 4d_1_–d_4_). The single dislocation indicated by the blue arrow in Fig. 4d_1_ were emitted from the specimen surface, as shown in Fig. 4d_2_. The single dislocation indicated by the yellow arrow moved to the right (Fig. 4d_1_–d_3_) and stopped at the high dislocation density wall (Fig. 4d_4_). Therefore, we concluded that the motions of the single dislocations are restricted by the high dislocation density wall.

In contrast to the single dislocation motions, the line patterns in Fig. 4 are seen to pass through the high dislocation density wall. Because deformation-induced martensite and twin plates are able to grow through dislocation cells and walls^21,22^, the observed line patterns possibly correspond to the group motion of partial dislocations. Since the fcc alloy used in this work has a low stacking fault energy of 20 mJ/m^2^ ^23–25^, twinning or fcc-to-hcp martensitic transformation with a group motion of Shockley partial dislocations can occur^21,26–29^. Therefore, we determined the Schmid factors for partial dislocation motion (summarized in Table 2). This table indicates that the group motion of partial dislocations most easily occurs on the (1 1 1) plane. The following are possible reactions derived from the Table 2:

3 $$\frac{1}{2}{[-101]}_{(111)}\,\to \,\frac{1}{6}{[-211]}_{(111)}+\frac{1}{6}{[-1-12]}_{(111)}$$

4 $$\frac{1}{2}{[-110]}_{(111)}\,\to \,\frac{1}{6}{[-211]}_{(111)}+\frac{1}{6}{[-12-1]}_{(111)}$$

**Table 2:** Schmid factors of the twinning system for the observational area of the ECCI.

Twinning plane	Twinning direction	Schmid factor
(1 1 1)	[−2 1 1]	0.43
	[−1 2 –1]	0.25
	[−1 –1 2]	0.18
(−1 1 1)	[2 1 1]	0.18
	[1 2 –1]	0.16
	[1 –1 2]	0.02
(1 –1 1)	[2 1 –1]	0.09
	[−1 –2 –1]	0.20
	[1 –1 –2]	0.29
(1 1 –1)	[2 –1 1]	0.07
	[1 –2 –1]	0.17
	[−1 –1 –2]	0.10

From these features, it is very possible to conclude that the ECCI detected the group motion of partial dislocations for twinning or fcc-to-hcp martensitic transformation.

In summary, we successfully characterized stress and lattice defect motion/formation behaviors in a bulk specimen using a scanning electron microscope-based technique. In particular, the dislocation-resolved ECCI technique was applied under loading conditions, which had never been reported before and demonstrated some patently clear advantages. Firstly, scanning electron microscope-based ECCI was performed in a bulk specimen and is, thereby, applicable for a specimen geometry used for normal tensile tests. Additionally, lattice strain and rotation were shown to alter ECCI, which facilitates the visualization of the variation in residual stress distribution, depending on remote stress conditions. Finally, ECCI enabled visualization of both dislocation glides and surface relief formation under loading. Therefore, since the observational area is at a grain-size-scale, the heterogeneous lattice defect motion and slip line formation in the crystal grain can be kinetically characterized using the approach reported herein.

However, the *in-situ* observations using ECCI were limited to the elastic regime to avoid distinct surface relief formation that could significantly disturb the quality of lattice images. Nevertheless, we believe that ECCI can be advantageously applied for analysis of more severe plastic deformations, provided that the orientation or the mechanical conditions are confined to induce in-plane plastic deformation.

## Methods

### Material preparation

We prepared an Fe15Mn10Cr8Ni austenitic steel (mass%). An ingot was prepared by vacuum induction melting. The ingot was forged and caliber rolled at 1373 K. Subsequently, the steel was solution-treated at 1273 K for 1 h followed by water quenching to suppress uncontrolled precipitation and segregation. The microstructure was fully austenitic prior to deformation, and showed deformation-induced ε martensitic transformations^17,20,23^. The solution-treated bar was cut into the desired shape by electrical discharge machining. The gauge shape of the tensile specimens was 2 mm^w^ × 1 mm^t^ × 10 mm^l^.

### Displacement holding experiment under SEM

Loading history schematics is shown in Fig. S1. Briefly, a specimen was firstly pre-deformed to 2% tensile strain (corresponding to 246 MPa: σ*) at room temperature with an initial strain rate of 5 × 10^−4^ s^−1^ (estimated from the cross-head speed); this was achieved using an *in-situ* tensile stage (TSL Solutions CO., Ltd) outside the scanning electron microscope (Merlin, Carl Zeiss). The strain was measured using a strain gauge. Then, the pre-deformed specimen was mechanically ground and polished, which reduced the specimen’s thickness by 30 µm. Afterwards, the pre-deformed specimen with the tensile stage was set into the field emission scanning electron microscope for performing the *in-situ* ECCI observations. ECCI was conducted in the region on the polished specimen surface, wherein a circular hole with a radius of 1 µm existed. The hole is associated with the presence of manganese oxide (MnO). The sample contains many spherical oxides with a diameter ranging from submicron to several microns. ECCI was operated at an acceleration voltage of 30 kV and a probe current of 2 nA at a working distance of 2.6 mm. When the surface orientation was optimized for a Bragg’s condition, the contrast in the resulting ECC image appears dark. Therefore, local residual stress can be visualized as a contrast gradation from dark to bright in the ECC image.

The pre-deformed specimen was held at a constant displacement for 24 min after reloading to 199 MPa (approximately σ* × 0.8), and subsequently for 281 min after reloading to 223 MPa (approximately σ* × 0.9) using the field-emission scanning electron microscope. During the displacement holding tests, we observed dislocation motion using ECCI. In this work, all ECCI was performed at 30 kV and 2 nA.

Thereafter, lattice defects and associated crystallographic features such as {1 1 1} traces were characterized using electron backscatter diffraction and secondary electron imaging. The electron backscatter diffraction measurement was conducted at 20 kV and 10 nA and a beam step size of 0.3 µm. The secondary electron imaging was conducted at an acceleration voltage of 10 kV, a probe current of 10 nA, and a working distance of 10.3 mm.

### Finite element method

The visualized residual stress distribution in the ECC image was simulated by calculating von Mises equivalent stress using the general-purpose finite element program ANSYS 17.0 (http://www.ansys.com/). The advantage of equivalent stress is expressing stress as a scalar, because the electron channeling contrast change via the residual stress is also a scalar criterion. The analysis conditions of the finite element method are shown in Fig. S2. Assuming that the observed hole is a hemisphere 1 µm in radius in the center of the specimen surface, one-quarter of the specimen was used in the analysis to take advantage of the symmetry and large deformations were applied. The material properties of the specimen are as follows: Young’s modulus *E* = 200 GPa, Poisson’s ratio = 0.27, and yield strength σ_Y_ = 162 MPa^20^. Tangent modulus *Y* after the yielding point was set to 1.90 GPa from the result of a tensile test as shown in Fig. S2c.

## Supplementary Information

Supplementary Information

Supplementary movie of Fig. 2c

Supplementary movie of Fig. 3a

Supplementary movie of Fig. 4c

Supplementary movie of Fig. 4d

## Data Availability

The datasets generated during and/or analyzed during the current study are available from the corresponding authors on reasonable request.
