# Supplementary material for: In-Situ Electron Channeling Contrast Imaging under Tensile Loading: Residual Stress, Dislocation Motion, and Slip Line Formation
Source: Sci Rep. 2020 Feb 14;10:2622. doi: 10.1038/s41598-020-59429-x (PMC7021723; doi:10.1038/s41598-020-59429-x)
Supplement: Supplementary file 1 — Supplementary Information [file 41598_2020_59429_MOESM1_ESM.docx]

*In-Situ* Electron Channeling Contrast Imaging under Tensile Loading: Residual Stress, Dislocation Motion, and Slip Line Formation

Keiichiro Nakafuji ^a^, Motomichi Koyama ^b^, Kaneaki Tsuzaki ^a,c*^

a Department of Mechanical Engineering, Kyushu University, Motooka 744, Nishi-ku, Fukuoka, 819-0395, Japan

b Institute for Materials Research, Tohoku University, 2-1-1 Katahira, Aoba-ku, Sendai, Miyagi 980-8577, Japan

c Elements Strategy Initiative for Structural Materials (ESISM), Kyoto University, Yoshida-honmachi, Sakyo-ku, Kyoto 606-8501, Japan

*corresponding author; e-mail address: tsuzaki.kaneaki.802@m.kyushu-u.ac.jp

Table 1. Schmid factors of the slip system for the observed grain. The incident beam direction was [0.51 -0.35 0.79] and the tensile direction was [-0.42 0.70 0.58].

| **Slip plane** | **Slip direction** | **Schmid factor** |
| --- | --- | --- |
| (1 1 1) | [0 1 -1] | 0.04 |
|  | [-1 0 1] | 0.35 |
|  | [1 -1 0] | 0.39 |
| (-1 1 1) | [0 -1 1] | 0.08 |
|  | [-1 0 -1] | 0.11 |
|  | [1 1 0] | 0.19 |
| (1 -1 1) | [0 1 1] | 0.28 |
|  | [1 0 -1] | 0.22 |
|  | [-1 -1 0] | 0.06 |
| (1 1 -1) | [0 -1 -1] | 0.16 |
|  | [1 0 1] | 0.02 |
|  | [-1 1 0] | 0.14 |

Table 2. Schmid factors of the twinning system for the observational area of the ECCI.

| **Twinning plane** | **Twinning direction** | **Schmid factor** |
| --- | --- | --- |
| (1 1 1) | [-2 1 1] | 0.43 |
|  | [-1 2 -1] | 0.25 |
|  | [-1 -1 2] | 0.18 |
| (-1 1 1) | [2 1 1] | 0.18 |
|  | [1 2 -1] | 0.16 |
|  | [1 -1 2] | 0.02 |
| (1 -1 1) | [2 1 -1] | 0.09 |
|  | [-1 -2 -1] | 0.20 |
|  | [1 -1 -2] | 0.29 |
| (1 1 -1) | [2 -1 1] | 0.07 |
|  | [1 -2 -1] | 0.17 |
|  | [-1 -1 -2] | 0.10 |
