# Supplementary figures and images for: In-Situ Electron Channeling Contrast Imaging under Tensile Loading: Residual Stress, Dislocation Motion, and Slip Line Formation
Source: Sci Rep. 2020 Feb 14;10:2622. doi: 10.1038/s41598-020-59429-x (PMC7021723; doi:10.1038/s41598-020-59429-x)

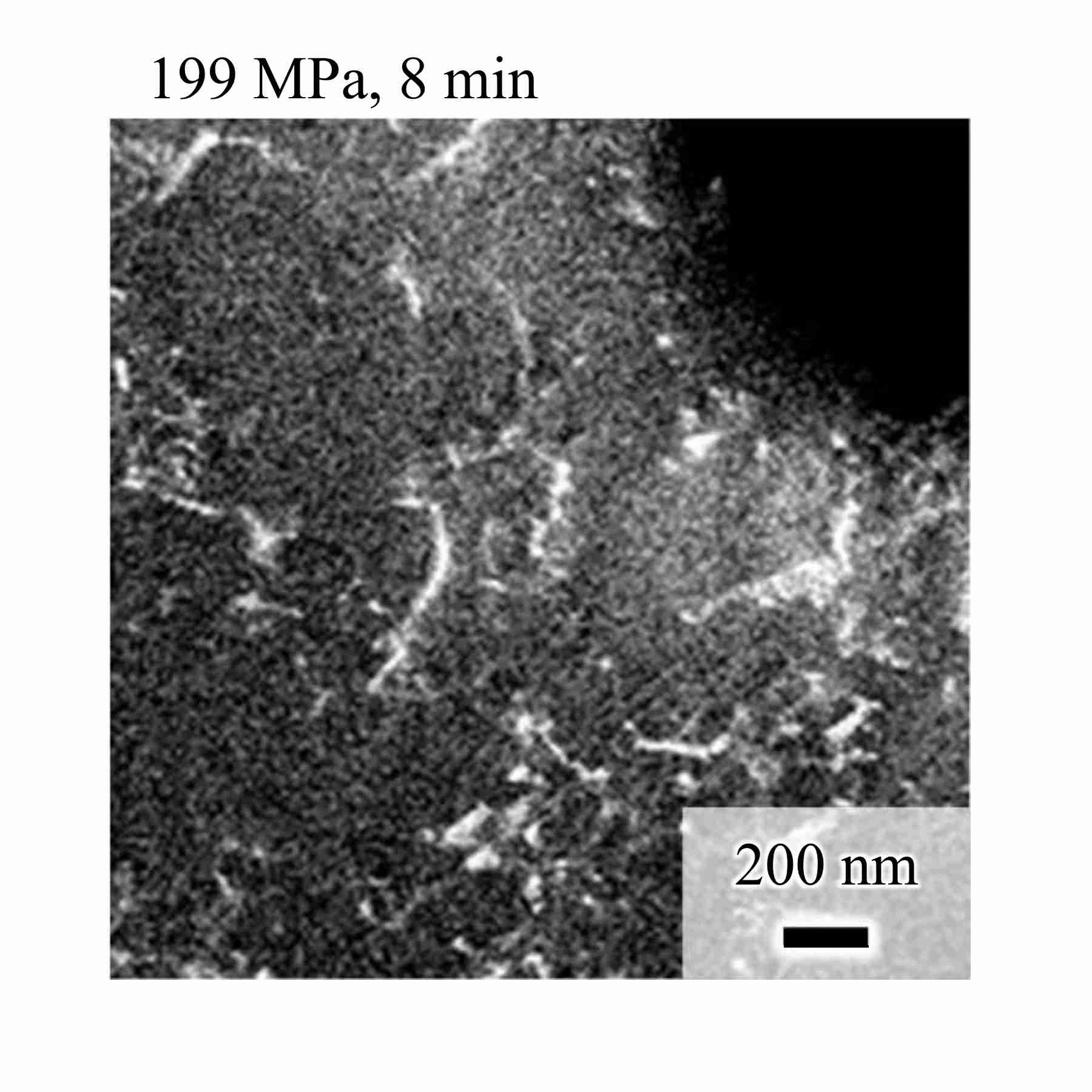

Supplement: Supplementary file 2 — Supplementary movie of Fig. 2c [file 41598_2020_59429_MOESM2_ESM.gif]

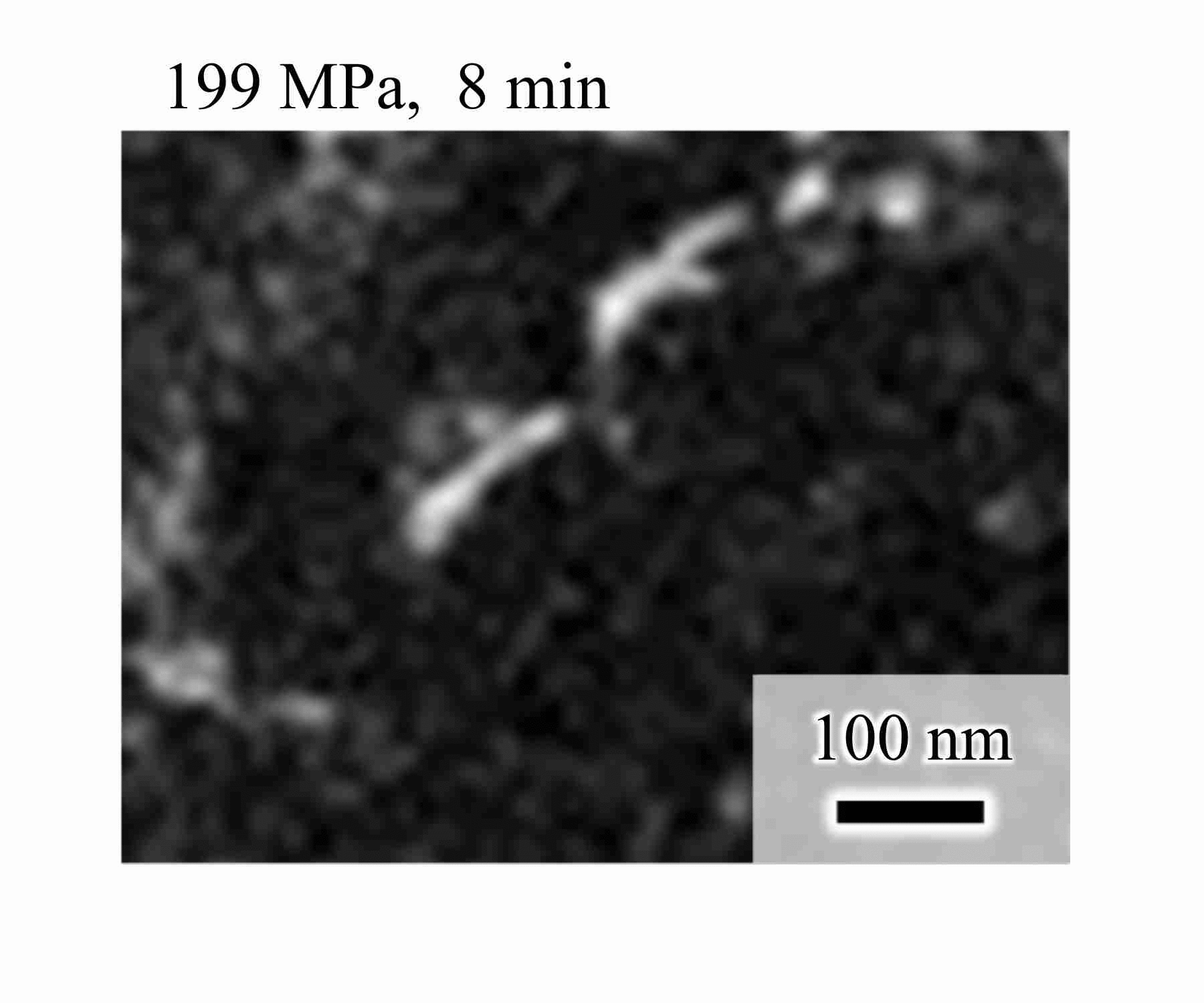

Supplement: Supplementary file 3 — Supplementary movie of Fig. 3a [file 41598_2020_59429_MOESM3_ESM.gif]

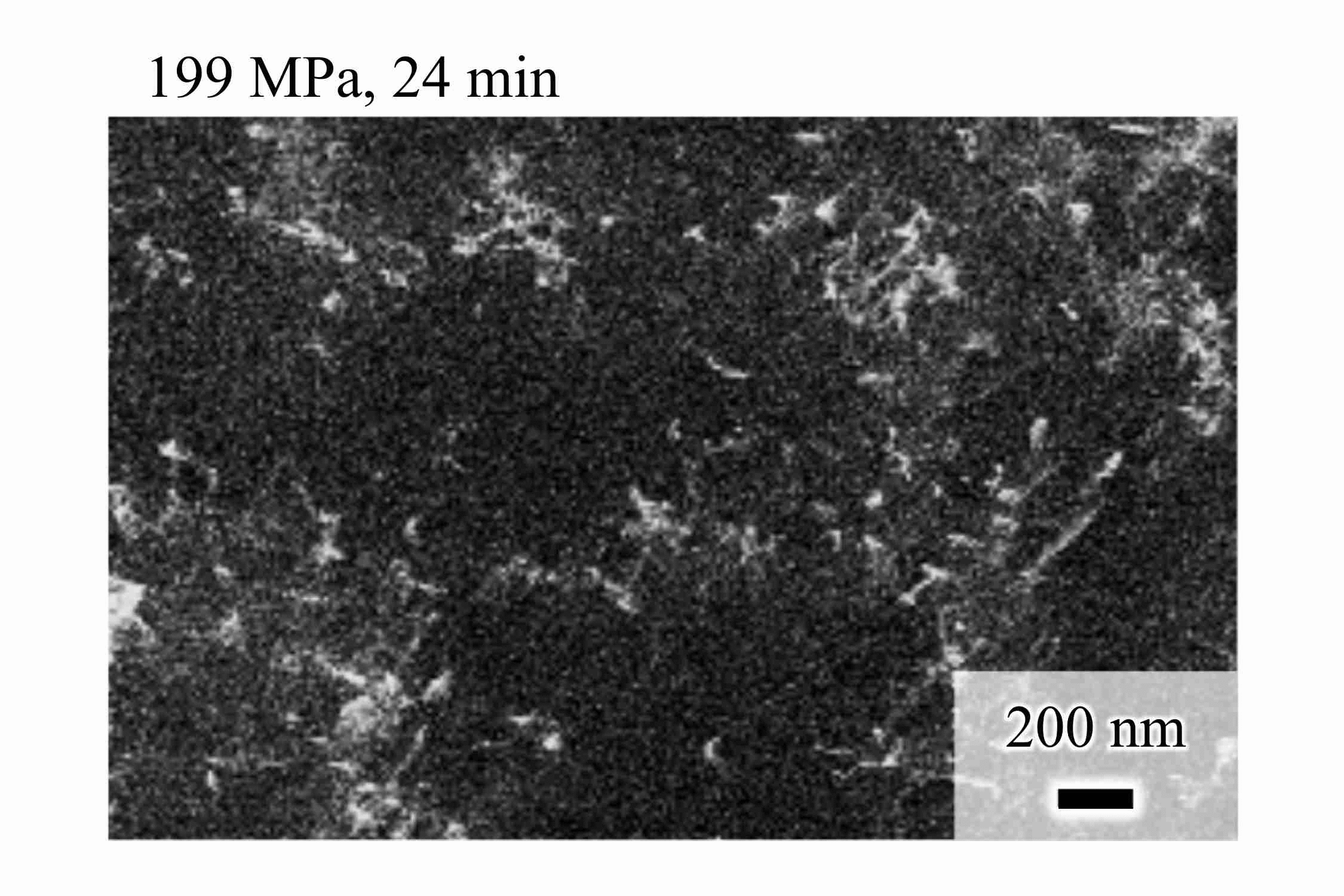

Supplement: Supplementary file 4 — Supplementary movie of Fig. 4c [file 41598_2020_59429_MOESM4_ESM.gif]

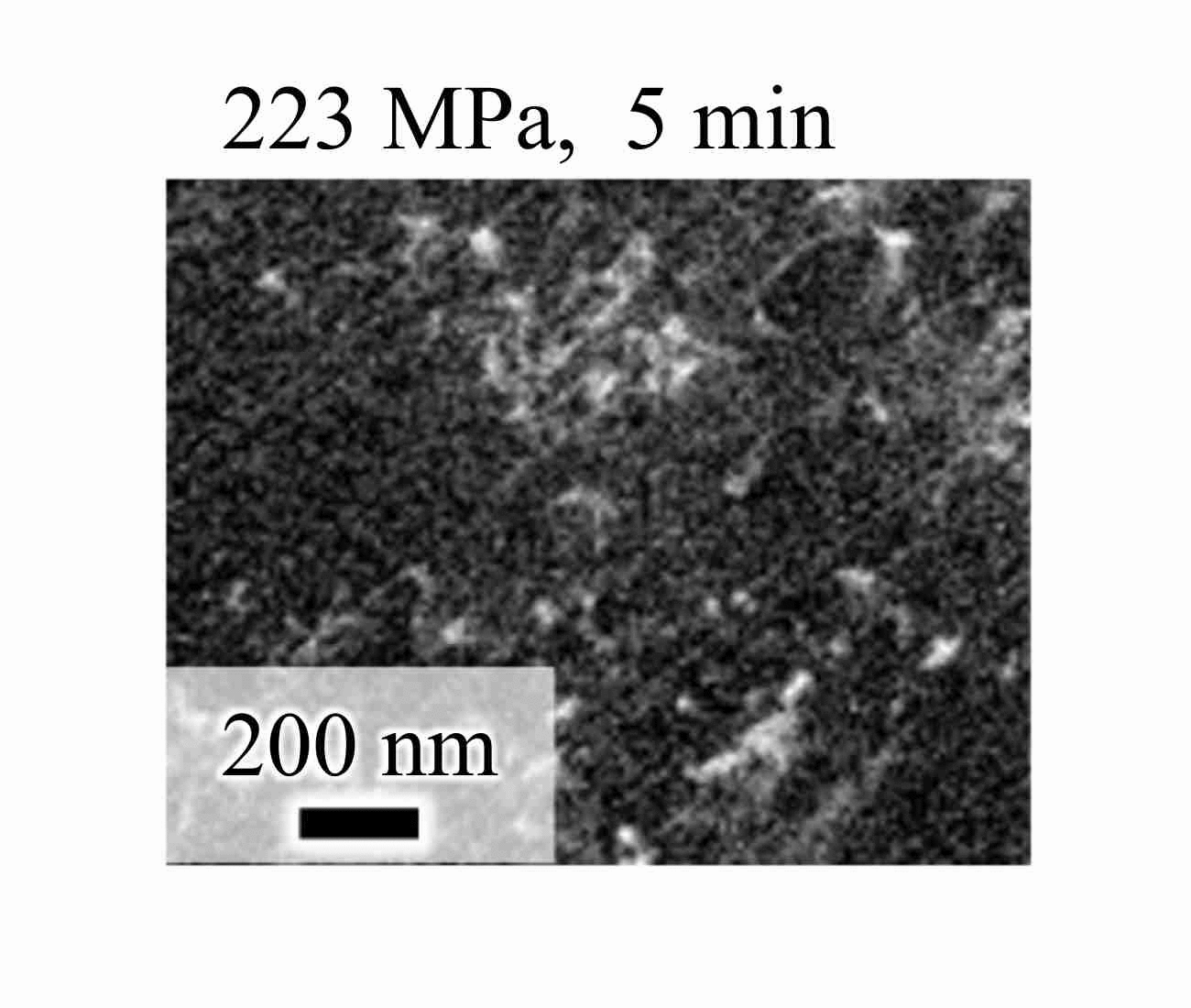

Supplement: Supplementary file 5 — Supplementary movie of Fig. 4d [file 41598_2020_59429_MOESM5_ESM.gif]
